# Socio-Demographic Determinants Associated with *Blastocystis* Infection in Arequipa, Peru

**DOI:** 10.4269/ajtmh.20-0631

**Published:** 2020-11-16

**Authors:** Renzo S. Salazar-Sánchez, Kasandra Ascuña-Durand, Jorge Ballón-Echegaray, Victor Vásquez-Huerta, Elí Martínez-Barrios, Ricardo Castillo-Neyra

**Affiliations:** 1 Laboratorio de Microbiología Molecular, Facultad de Medicina, Universidad Nacional de San Agustín, Arequipa, Peru;; 2 One Health Unit, Zoonotic Disease Research Lab, School of Public Health and Administration, Universidad Peruana Cayetano Heredia, Lima, Peru;; 3 Departamento de Microbiología y Patología, Facultad de Medicina, Universidad Nacional de San Agustín, Arequipa, Peru;; 4 Department of Biostatistics, Epidemiology and Informatics, Perelman School of Medicine of the University of Pennsylvania, Philadelphia, Pennsylvania

## Abstract

*Blastocystis* is one of the most common protozoa in the human gut and a zoonotic organism related to unsanitary living conditions. This protozoon shows a broad distribution, unclear symptomatology, and undefined pathogenicity. In Peru, studies report the presence of *Blastocystis* in many regions, but the highest prevalence levels are reported in Arequipa. The aim of this study was to link *Blastocystis* infection with social determinants of health. We recruited and surveyed 232 infected and uninfected participants from houses with at least one *Blastocystis*-infected person. All samples were concentrated by spin concentration method in saline solution, examined by wet mount under light microscopy and confirmed with methylene-stained stool smear. We found a human *Blastocystis* prevalence of 51.3% in the study sample. We also found statistical associations between *Blastocystis* infection and peri-urban location in the city as well as the use of alternative non-domiciliary water supplies, suggesting these are risk factors for human *Blastocystis* infection.

## INTRODUCTION

Intestinal parasitic infections are one of the most common public health problems, affecting more than two million people around the world.^1,2^ They are mainly found in areas with poor health and sanitary conditions, limited access to safe drinking water, inadequate disposal of human feces,^3,4^ and low levels of access to healthcare facilities.^5^ Among many helminths and protozoa with the potential to inhabit the human gut, *Blastocystis* is one of the most commonly identified organisms during stool examination.^6^

*Blastocystis* has a worldwide distribution and is the most commonly isolated microorganism in parasitological surveys.^6^ Despite being so ubiquitous and being discovered more than a 100 years ago,^7^ little is known about their pathogenicity, genetic diversity, transmission dynamics (including zoonotic and zooanthroponotic transmission), therapeutic options, and treatment efficacy.^8,9^ The role of *Blastocystis* in human disease remains controversial.^10^ Its presence in symptomatic and asymptomatic patients is difficult to explain; therefore, some report it as a pathogen, whereas others regard it as a commensal.^11,12^

Many risk factors are associated with *Blastocystis* infection, including to be 6- or 7-year-old child,^13^ male,^14,15^ and flooding of the home. The use of a latrine as compared with flush toilets is also associated with *Blastocystis* infection likely because of its relation to poor hygiene,^14,16^ water-borne transmission, and the lack of access to treated drinking water.^16,17^ By contrast, other studies mentioned that sociodemographic factors such as age, gender, water quality, disposition of excreta, place of residence, number of children in the house, monthly income, type of property, floor type, wall type, availability of public services, handwashing habits, and garbage disposal were not associated with *Blastocystis* infection.^18^ Domestic and wild animals are considered an important source of *Blastocystis* transmission.^8,18^ However, previous studies suggest that livestock animals are not the main contributor of human infections.^19^ Carnivores, reptiles, and insects do not seem to be important sources of infection either.^18^

Most of these risk factor studies have been conducted in Asia and Europe, with few studies focusing on Latin American countries. In Peru, the presence of *Blastocystis* has been reported in many regions, mainly in parasitological surveys carried out in schoolchildren.^20–22^ The most common association with *Blastocystis* infection reported among positive study sites was poor sanitary conditions.^23,24^ Among the 24 regions in Peru, the highest prevalence has been reported in Arequipa.^23^ These previous studies did not aim to find factors associated with the high prevalence of infection in Arequipa or evidence of their association with symptomatology and gastrointestinal illnesses in infected cases.

Because of *Blastocystis*’ disputed role as a pathogenic agent and the scarcity of Peruvian studies on factors associated with infection and symptomatology, this study aimed to identify individual and household-level factors associated with *Blastocystis* infections, and to add new evidence for the understanding of the complex epidemiology of this controversial intestinal protozoon. We studied *Blastocystis* infection in humans and animals across different levels of urbanization in Arequipa, Peru, which represents one of the first attempts in the region to apply the One Health approach to this microorganism. One Health is an approach that recognizes the connection among human, animal, and environmental health.^25^

## MATERIALS AND METHODS

### Ethical statement.

The Institutional Review Boards of the Universidad Peruana Cayetano Heredia approved the protocol of this study, identification number 18006. Before collecting any data, all participants provided written informed consent. Minors provided verbal and/or written informed assent, and their parents provided written informed consent. We included participants from any age who were not taking any antimicrobial or antiparasitic treatment at the time of stool sample collection or the prior 7 days. All participants completed an epidemiological survey to assess clinical and sanitary living conditions. Participants’ questions were solved at any moment.

### Study site.

**Figure 1.**
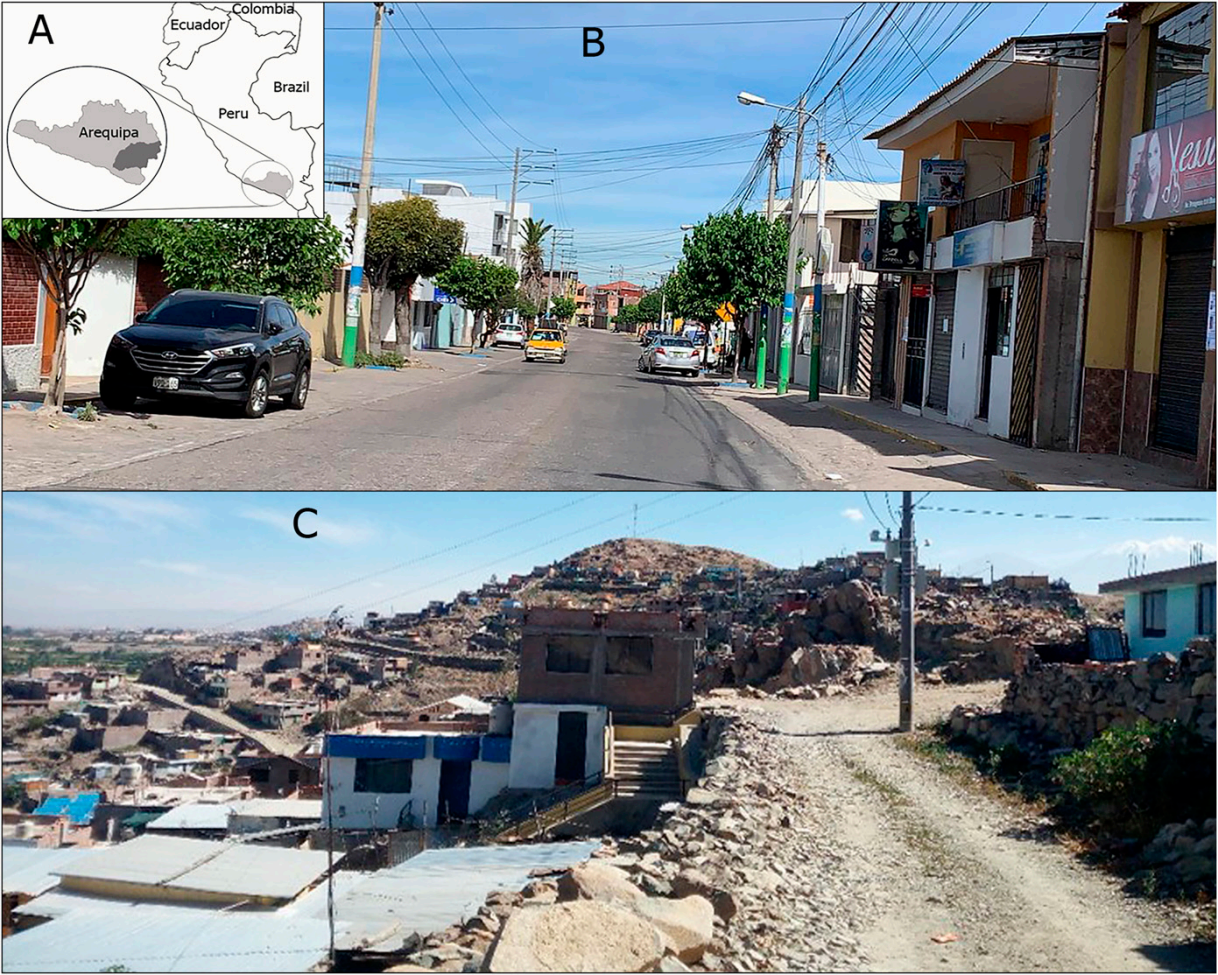
Study site. (**A**) Map of Arequipa in Peru. (**B**) Urban locality with adequate sanitary conditions and access to public facilities. (**C**) Peri-urban locality with limited sanitary conditions. This figure appears in color at www.ajtmh.org.

This study was conducted in Arequipa city, which is located in the south highlands with a population size around a million people.^26^ Arequipa city comprises peri-urban and urban areas, which have distinct migration histories, and continuous growth from the center to the periphery.^27^ In the context of this centrifugal expansion, the peri-urban communities are younger than the urban communities and are farther from the center of the city.^27^ There are also social differences between the two types of communities: peri-urban areas tend to have limited basic services such as electricity, water, sanitation, health, and education, whereas urban locations have all these services more readily available (Figure 1).

### Study population and participants’ selection.

**Figure 2.**
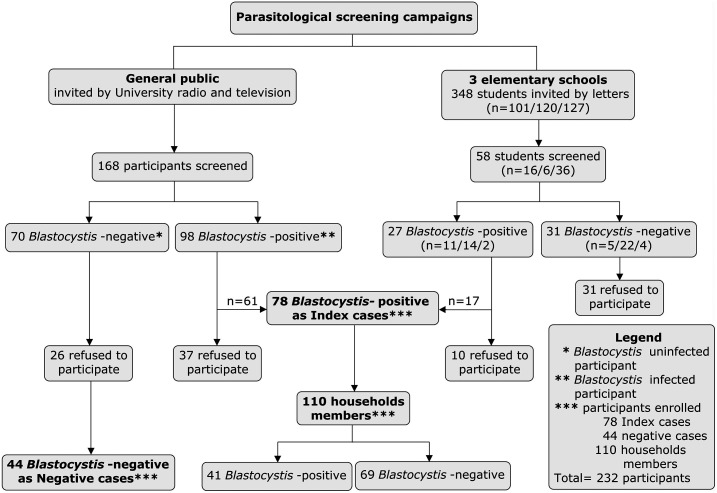
Diagram of recruitment campaigns and participant’s enrollment and distribution.

To identify research participants, we carried out two free parasitological screening campaigns. The first campaign was advertised on the radio (Radio Universidad Arequipa) and television (TV UNSA) for 3 weeks in January 2019, inviting any resident of Arequipa to participate in the campaign. The collection of samples, analysis, and reporting of results occurred in the Molecular Microbiology Laboratory, Universidad Nacional de San Agustín de Arequipa in Peru between January and March of 2019. One hundred sixty-eight people participated in the parasitological screening campaign, 102 tested positive for *Blastocystis* infection, and 66 were negative. We invited all 168 participants to follow-up on their household members, 61 of the 102 *Blastocystis*-positive participants and 44 of the 66 *Blastocystis*-negative participants accepted the invitation, and their household members were contacted to participate in the study. Detailed information of participants of the campaigns and recruitment is included in Figure 2.

For the purpose of this study, index cases were defined as any *Blastocystis*-positive participant identified during the initial screening campaigns and negative cases who were negative to any intestinal protozoon and parasite. To recruit household members of index cases, we communicated by phone or in person with index cases or parents from index cases to coordinate a date for visiting their home. Then, we invited household members to participate in the study with a free parasitological screening for them and their animals. All results were reported to participants in person 2 days after taking the stool sample. The research team medical doctor provided with antiparasitic treatment to people who were infected by any kind of intestinal protozoan.

### Sample collection and laboratory diagnosis.

All samples were collected by each participant in a sterilized plastic wide-mouth flask without additives. We instructed participants to avoid mixing with urine or water and wash hands with soap after collection. Instructions were given in person in a short letter.

To determine the presence of trophozoites and other protozoa stages, we applied a rapid spin concentration method with saline solution and examined the pellet with Lugol solution using a wet mount slide under light microscopy at ×400 magnification. The results were confirmed with blue methylene-stained stool smear at ×1,000 magnification.^28^ All samples were aliquoted into cryovials and stored at −80°C.

### Statistical analysis.

Independent associations between *Blastocystis* infection and categorical demographic and sanitary conditions were analyzed with chi-square and Fisher’s exact test. A multivariable logistic regression model was built to identify adjusted risk factors for *Blastocystis* infection. All analyses were performed using R 3.6.2.^29^

## RESULTS

We analyzed stool samples from 337 people and enrolled 232 participants during the campaigns. Participants were classified into 78 index cases, 110 household members (41 *Blastocystis*-positive and 69 *Blastocystis*-negative participants), and 44 negative cases. The age range of participants was 1–81 years (mean = 37, SD = 21.8), and the percentage of female participants was 55.6% (*n* = 129). One hundred twenty-two participants are from urban locations, 101 from peri-urban locations, and nine came from rural areas. The education level of participants was 54 in elementary school, 44 high school, and 128 with college or advanced degrees. Professionally, our sample included 39 homemakers, 74 students, 42 employees, 66 independents contractors, and five retirees.

**Figure 3.**
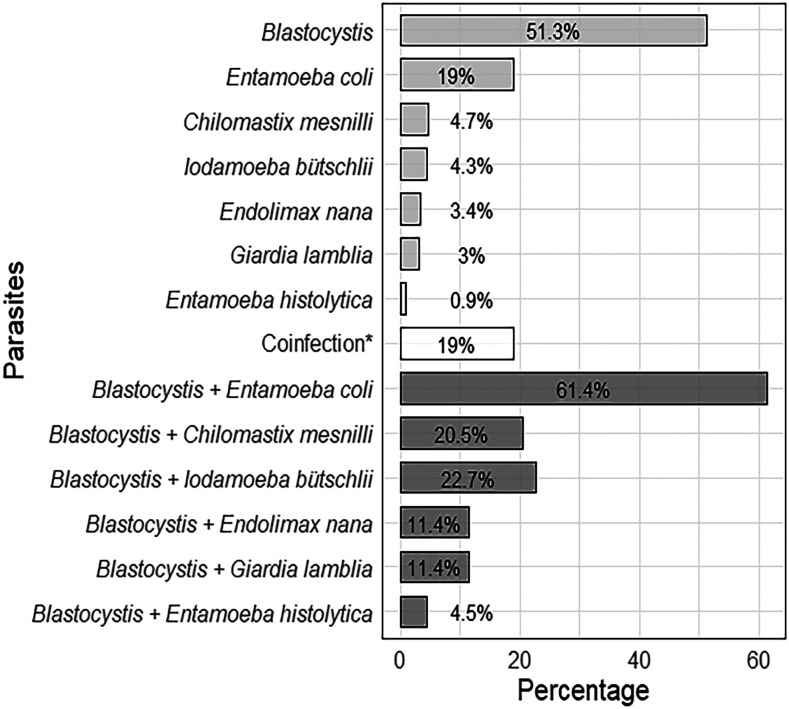
Percentage of intestinal parasites and *Blastocystis* coinfection in the study sample from Arequipa, Peru, in 2019 (*n* = 232). *Includes only *Blastocystis* coinfection with other parasites (*Blastocystis* coinfection in dark gray).

**Figure 4.**
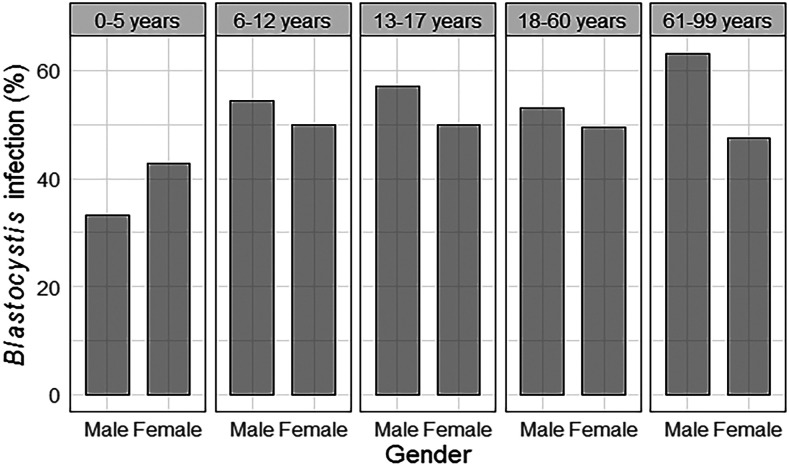
Percentage of *Blastocystis* infection associated with age and gender.

We identified seven species of intestinal protozoa, including two pathogenics. The prevalence of *Blastocystis* in the study sample was 51.3%, with a coinfection percentage of 19%. The second most prevalent protozoon was *Entamoeba coli* (19%), as described in Figure 3. To explore the relationship between gastrointestinal symptomatology and *Blastocystis* infection, we included only participants with *Blastocystis* single infection (*n* = 46) and all participants who were negative to *Blastocystis* and any other protozoa and parasite (*n* = 65). Symptomatic participants showed no significant differences in *Blastocystis* prevalence (41.4%) compared with asymptomatic participants (49.1%). The two most frequent symptoms were flatulence and abdominal pain (44% and 36%), and the less frequent was irritable bowel syndrome (IBS) (5.3%). Statistical analysis (*n* = 167) shows that symptomatology was not significantly different between *Blastocystis*-positive and *Blastocystis-*negative participants. Specific symptoms were also not significantly associated with *Blastocystis* infection (Table 1). We observed a higher percentage of *Blastocystis* infection in male participants across all age-groups, except in children younger than 5 years, and there was a positive trend of infection associated with age (Figure 4).

**Table 1:** Association between gastrointestinal symptoms and *Blastocystis* infection status

	*Blastocystis* infection, *n* (%)		*P*-value
Variable	Uninfected	Infected	
	(*n* = 44)*	(*n* = 73)†	
Abdominal pain	36 (39.1)	27 (36.0)	0.799‡
Nausea and vomiting	14 (15.2)	11 (14.7)	1‡
Flatulence	46 (50.0)	33 (44.0)	0.537‡
Constipation	28 (30.4)	16 (21.3)	0.25‡
Diarrhea	27 (29.3)	17 (22.7)	0.425‡
IBS	3 (3.3)	4 (5.3)	0.702§

* Number of *Blastocystis-*uninfected participants includes 44 negative cases and 21 household members with negative result to *Blastocystis* and any other protozoa and parasite.

† Number of *Blastocystis*-infected participants corresponds to participants with *Blastocystis* single infection.

‡ Chi-square test.

§ Fisher’s exact test.

To set the statistical analysis for comparisons, we grouped our variables into sanitary variables and demographic variables, that include hygienic habits. The first group includes variables related to sanitary conditions such as location, water supply, body-waste disposal, presence of animals, and vectors. For all the analyses, we dropped participants from the rural location, and collapsed the water supply category into domiciliary tap water and other water supply that included public standpipe, water tank, and water well. Body-waste disposal was categorized into use of flush toilets and use of latrine, which also included silo (a basic and precarious latrine). Table 2 presents the statistical analysis for independent association of sanitary conditions, which was performed at the house level (*n* = 117), including houses from index cases and negative cases. We observed a higher percentage of *Blastocystis* infection in peri-urban areas than in urban areas (*P* < 0.001). Also, there were significant associations among *Blastocystis* infection and body-waste disposal, and presence of rabbits and rodents. Other variables show no statistical association with *Blastocystis* infection in homes from index cases and negative controls.

**Table 2:** Sanitary characteristics of houses from index cases (*Blastocystis*-infected participants) and negative cases (*Blastocystis*-uninfected participants)

	*Blastocystis* infection, *n* (%)		*P*-value
Variable	Uninfected	Infected	
	(*n* = 44)*	(*n* = 73)†	
Location			
Urban	37 (84.1)	31 (42.5)	**0.0001**‡
Peri-urban	7 (15.9)	42 (57.5)	
Water supply			
Domiciliary tap water	43 (97.7)	66 (90.4)	0.255§
Other water supply	1 (2.3)	7 (9.6)	
Body-waste disposal			
Piped sewer system	43 (97.7)	63 (86.3)	**0.051**‡
Latrine	1 (2.3)	10 (13.7)	
Presence of dog	24 (54.5)	42 (57.5)	0.902‡
Presence of cat	10 (22.7)	24 (32.9)	0.337‡
Presence of guinea pig	8 (18.2)	17 (23.3)	0.675‡
Presence of rabbit	1 (2.3)	14 (19.2)	**0.018**‡
Presence of poultry	11 (25.0)	18 (24.7)	1‡
Presence of flies	31 (70.5)	50 (68.5)	0.987‡
Presence of cockroach	6 (13.6)	16 (21.9)	0.386‡

Bolding highlights significant *P*-values.

* *n* corresponds to negative cases (participants with negative result to *Blastocystis* and any other protozoa and parasite).

† *n* corresponds to index cases (first *Blastocystis*-positive participant identified during the initial screening campaigns).

‡ Chi-square test.

§ Fisher’s exact test.

The second group included variables related to demographic and hygienic habits of participants such as gender, age, education level, economic activity, food consumption place, kind of water consumption, handwashing habits, and cleanliness of hands and nails at the time of the survey. The economic activity represents participants’ occupations, where different professions were identified and summarized as students, homemakers, employees, independent contractors, and retired. We performed statistical analysis at the individual level (*n* = 155) with *Blastocystis*-infected and uninfected participants from houses with at least one *Blastocystis*-positive participant to determine intra-household factors associated with *Blastocystis* transmission. We do not include the few participants whose responses to hygienic habit questions were “sometimes.” The outcome is presented in Table 3, where no variable shows a significant association with *Blastocystis* infection.

**Table 3:** *Blastocystis* infection status associated with characteristics of participants at individual level

Variable	*Blastocystis* infection,* *n* (%)		*P*-value
	Uninfected	Infected	
	*n* = 68	*n* = 87	
Gender			
Female	42 (61.8)	44 (50.6)	0.219†
Age-group (years)			
0–5	4 (5.9)	4 (4.6)	0.815‡
6–12	13 (19.1)	17 (19.5)	
13–17	4 (5.9)	6 (6.9)	
18–60	41 (60.3)	47 (54.0)	
61–99	6 (8.8)	13 (14.9)	
Education level			
Elementary	17 (25.8)	21 (24.7)	0.918†
High school	17 (25.8)	20 (23.5)	
University	32 (48.5)	44 (51.8)	
Economic activity			
Student	27 (40.9)	31 (38.3)	0.925†
Homemaker	11 (16.7)	13 (16.0)	
Employees	8 (12.1)	13 (16.0)	
Independent contractor	20 (30.3)	24 (29.6)	
Food consumption place			
House	45 (66.2)	61 (70.1)	0.636‡
Restaurant	1 (1.5)	0	
House and restaurant	15 (22.1)	20 (23.0)	
House and kiosk	7 (10.3)	6 (6.9)	
Kind of water consumption			
Boiled water	54 (79.4)	68 (78.2)	1‡
Tap water	4 (5.9)	6 (6.9)	
Both	10 (14.7)	13 (14.9)	
Do not wash hands after using bathroom	6 (8.8)	7 (8.0)	1†
Do not wash hands before cooking	13 (19.1)	22 (25.3)	0.4727†
Do not wash hands before eating	11 (16.7)	9 (10.5)	0.3794†
Do not wash hands after touching animals	21 (31.3)	31 (35.6)	0.6994†
Had dirty hands at the time of survey	13 (19.1)	21 (24.1)	0.5796†
Had dirty nails at the time of survey	21 (30.9)	32 (36.8)	0.55†

* The analysis includes only index cases and their household members.

† Chi square test.

‡ Fisher exact test.

Bivariate analyses highlighted that location (odds ratio [OR] = 7.2), other water supply (OR = 4.6), use of latrine (OR = 6.8), and presence of rabbits (OR = 10.2) were statistically associated with *Blastocystis* infection. To set up the multivariate analysis, we included variables yielding a *P*-value less than or equal to 0.2 in the bivariate analysis and performed a stepwise approach to determine interaction and effect of independent variables on *Blastocystis* infection. Multivariate analysis showed that the only variables that affect *Blastocystis* infection were living in peri-urban areas (OR = 3.7), having or using a different water supply than domiciliary tap water (OR = 19.3), and presence of dogs (OR = 10.2); the most significant association was with domiciliary water supply (Table 4). Other variables in the analysis showed no significant association with *Blastocystis* infection, mainly variables related to demographic and hygienic habits.

**Table 4:** Bivariate and multivariate analysis of *Blastocystis* infection risk factors

Variable	Bivariate logistic regression			Multivariate logistic regression		
	OR*	95% CI	*P*-value	OR†	95% CI	*P*-value
Peri-urban location	7.2	3.0–19.5	**< 0.001**	3.7	1.4–10.8	**0.012**
Water supply: other water supply	4.6	0.8–86.8	0.101	19.3	1.5–425.2	**0.038**
Body-waste disposal: latrine	6.8	1.2–127.6	**0.024**	0.1	0–0.8	**0.040**
Presence of dog	1.1	0.5–2.4	0.752	10.2	2.0–59.7	**0.006**
Presence of cat	1.7	0.7–4.1	0.236	–	–	–
Presence of guinea pig	1.4	0.6–3.7	0.511	–	–	–
Presence of rabbit	10.2	1.9–188.4	**0.003**	2.0	0.6–7.3	0.258
Presence of poultry	1.0	0.4–2.4	0.967	–	–	–
Presence of flies	0.9	0.4–2.0	0.824	–	–	–
Presence of cockroach	1.8	0.7–5.3	0.258	–	–	–
Female participants	0.6	0.3–1.2	0.163	0.6	0.3–1.3	0.178
Age-group (years)						
13–17	1.5	0.2–10.4	0.672	–	–	–
18–60	1.1	0.3–5.1	0.853	–	–	–
61–99	2.2	0.4–12.4	0.370	–	–	–
Education						
High school	1.0	0.4–2.4	0.916	–	–	–
University	1.1	0.5–2.4	0.789	–	–	–
Food consumption place						
House and restaurant	1.0	0.5–2.2	0.967	–	–	–
House and kiosk	0.6	0.2–2.0	0.437	–	–	–
Kind of water consumption						
Tap water	1.2	0.3–4.9	0.794	–	–	–
Boiled and tap water	1.0	0.4–2.6	0.945	–	–	–
Do not wash hands after using bathroom	1.1	0.3–3.5	0.863	–	–	–
Do not wash hands before cooking	0.7	0.3–1.5	0.359	–	–	–
Do not wash hands before eating	1.7	0.7–4.5	0.265	–	–	–
Do not wash hands after touching animals	0.8	0.4–1.6	0.576	–	–	–
Had dirty hands at the time of survey	1.3	0.6–3	0.452	–	–	–
Had dirty nails at the time of survey	1.3	0.7–2.6	0.441	–	–	–

Bolding highlights significant *P*-values.

* Unadjusted OR values.

† Adjusted OR values.

## DISCUSSION

The controversy surrounding *Blastocystis* in recent decades has resulted in increased interest in developing detailed studies focused on determining whether or not the protozoon is pathogenic or beneficial to infected humans.^9,30^ Most previous studies in Arequipa focused on reporting intestinal parasites’ prevalence in elementary schoolchildren. Those reports identified *Blastocystis* as a recurrent protozoon in this population, although they did not link epidemiological data to clinical outcomes. This is the first study that aimed to use the One Health approach to identify social determinants and individual factors associated with *Blastocystis* infection in Arequipa, Peru.

The prevalence of *Blastocystis* infection found in our study was similar to other values reported in previous studies in Peru.^20–22^ However, it was in the lower boundary of the range previously reported in Arequipa (48.3–81.9%).^23,24^ This lower prevalence can be explained by the fact that previous studies in Arequipa were focused on schoolchildren, and we included all age-groups. We observed prevalence of *Blastocystis* infection above 50% in schoolchildren and lower values in people ages 60 years or older and infants. However, *Blastocystis* was the most prevalent protozoon in this study sample, which is similar to other studies around the world.^31,32^ Likewise, we found no significant difference in the infection rate according to age or gender, a trend which has been reported in other studies.^33,34^ Our results counter findings from rural populations of rural Honduras where age was associated with the amount of *Blastocystis* DNA detected.^35^ Also, in rural populations of Brazil, *Blastocystis* infection was linked to being female and being homemakers.^15^

This study highlights living in peri-urban communities as one of the most important risk factors for the transmission of *Blastocystis* in humans. This is supported by a WHO report on social determinants of health,^36^ which described how peri-urban areas often have unhealthy sanitary conditions linked to poverty that facilitate health problems and increase rates of infectious diseases. These conditions are quite similar to rural areas, the origin of local migration to peri-urban areas of Arequipa.^37^

We identified that water supply is the other main risk factor for *Blastocystis* transmission in peri-urban locations. Because of the limited access to domiciliary tap water, people in peri-urban areas often consume water from standpipes, water tanks, or wells. Some studies have reported similar results, pointing to the lack of piped water and public sewage^38^; meanwhile, other studies were focused on areas with poor hygiene and sanitation, and facilities with a high prevalence of *Blastocystis*,^14,39^ whereas others studies did not find this association.^40^ *Blastocystis* has been reported in bodies of water in the veterinary literature^39,41^ and in contaminated water, which has been reported as a source of infection.^13^

The presence of animals is a common characteristic in peri-urban locations in Arequipa, where community members raise animals as a food resource, a rural custom brought to city because of migration.^37^ We found that the presence of dogs and rabbits is associated with *Blastocystis* infection in humans. This finding suggests that animals can play a key role in *Blastocystis* zoonotic transmission into infected homes and act as secondary reservoirs for *Blastocystis*.^42^ Furthermore, other studies in Latin America detected *Blastocystis* sp. in wild animal species, suggesting other cycles of transmission and potential reservoir species.^43^ Therefore, a One Health approach may be helpful in understanding the epidemiology of *Blastocystis*. This approach is also supported by previous studies that reported the occurrence of *Blastocystis* infection in different animal species such as poultry, cattle, pigs, and dogs.^44,45^ Similar associations to these animal species have been reported in endemic zoonotic disease in Arequipa.^46^

This study provides an overview sociodemographic determinant associated with *Blastocystis* infections; however, we could not determinate the commensal or pathogenic role of *Blastocystis* in humans. Some limitations of our study are the different procedures for participant recruitment and low participation in our campaigns. We were also unable to enroll all household members of every index case to estimate the household-level burden and potential factors associated with the level of infection within houses. It is also possible that the low response rate introduced selection bias in our study. Furthermore, our study design was cross-sectional; therefore, it is possible that *Blastocystis* infection is transient and with a high rate of coinfection. Finally, the sampling we used for the university-based campaign was not random, and some of the variables we analyzed are correlated. Future studies would benefit from improved recruitment strategies, including rural areas, and conducting longitudinal studies in humans and their animals to assess changes in *Blastocystis* infection status and potential transmission pathways.

## CONCLUSION

We found that human *Blastocystis* infection is associated with a group of factors that are found in peri-urban environments in the city of Arequipa, such as using an alternative not domiciliary water supply and using latrines for human waste body-waste disposal. The role of animals such as dogs and rabbits further elucidated to understand if they act as reservoirs or recipients of the protozoon (zooanthroponosis). We did not find a link between *Blastocystis* infection and gastrointestinal symptomatology in our study, so that, we cannot rule out the possibility that *Blastocystis* does have pathogenic potential, and they were not present in our study area or were present in very low frequencies.
